# Case Report: Rare co-occurrence of NMOSD and capillary leak syndrome treated with satralizumab

**DOI:** 10.3389/fimmu.2026.1684380

**Published:** 2026-03-04

**Authors:** Juanjuan Chen, Guogao Zhang, Qi Weng, Zhijian Lin, Xin Shi, Jun Hu

**Affiliations:** Department of Neurology, Peking University Shenzhen Hospital, Shenzhen, China

**Keywords:** autoimmune disease, case report, interleukin-6, NMOSD, systemic capillary leak syndrome

## Abstract

This report describes a rare case of neuromyelitis optica spectrum disorder (NMOSD) complicated by systemic capillary leak syndrome (SCLS) and reviews the literature to examine the clinical features, pathogenesis, and therapeutic implications of autoimmune disease–associated SCLS. A 20-year-old woman with NMOSD developed sudden-onset SCLS, presenting with hypotension, hemoconcentration (hematocrit 58.7%), hypoalbuminemia (26 g/L), and pulmonary edema after initial immunotherapy. Cardiogenic and septic shock, as well as pulmonary embolism, were excluded, leading to a diagnosis of SCLS. Intensive treatment with albumin replacement, thoracic drainage, and intravenous immunoglobulin (IVIG, 0.4 g/kg/day) stabilized her condition. Maintenance therapy with satralizumab, an anti–interleukin-6 receptor monoclonal antibody, achieved sustained remission over 1 year. A review of 12 cases (including this case) identified autoimmune diseases—most commonly Sjögren’s syndrome (41.7%) and NMOSD (16.7%)—as frequent SCLS comorbidities. Infections (33.3%) and autoimmune flares (25%) were the most common triggers. Multimodal therapy combining glucocorticoids and IVIG, with or without additional immunosuppressants, resulted in clinical improvement in 75% of cases. This report emphasizes that SCLS is a life-threatening complication of autoimmune diseases, particularly NMOSD. Autoimmune disease relapses and infections are common precipitating factors. Prompt diagnosis and intervention are critical. Satralizumab warrants further investigation as a potential therapeutic option for this rare comorbidity.

## Background

Neuromyelitis optica spectrum disorder (NMOSD) is a rare autoimmune disease of the central nervous system, primarily characterized by severe inflammatory demyelination of the optic nerves and spinal cord. It is strongly associated with autoantibodies against aquaporin-4 (AQP-4), a water channel protein on astrocytes, which plays a pivotal role in its pathogenesis (1).

Systemic capillary leak syndrome (SCLS), also known as Clarkson’s disease, is a rare but potentially fatal disorder involving recurrent episodes of severe hypotension, hypovolemia, and interstitial edema. The clinical presentation of SCLS comprises acute vascular collapse resulting from a sudden and transient increase in vascular permeability. This pathological process permits plasma leakage from the intravascular compartment into the interstitial space, leading to hemoconcentration, hypoalbuminemia, and tissue swelling. Subsequent reduction in effective circulating blood volume may cause severe circulatory shock and multi-organ dysfunction, often requiring urgent resuscitative measures (2, 3).

The co-occurrence of NMOSD and SCLS is exceedingly rare, presenting complex clinical manifestations that frequently culminate in severe outcomes. This report details a case of concomitant NMOSD and SCLS. The objectives of this study were threefold: (1) to delineate the clinical course and management of NMOSD complicated by SCLS; (2) to comprehensively review published cases of autoimmune disease-associated SCLS; and (3) to identify potential therapeutic targets for this rare comorbidity.

## Case report

**Figure 1:**
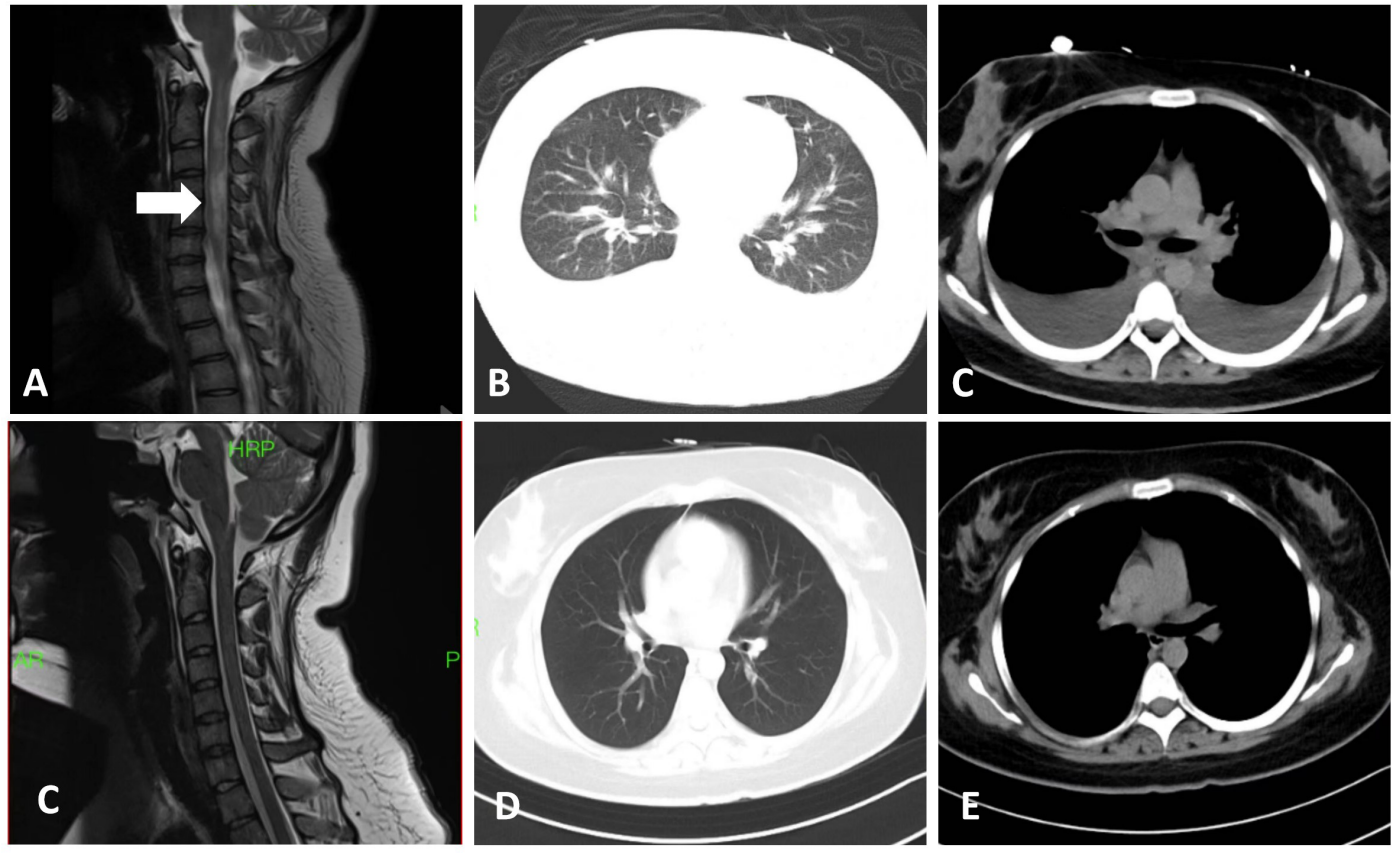
Imaging findings in our patient. **(A)** Cervical magnetic resonance image (T2-weighted) showing abnormal hyperintensity in the spinal cord spanning the C2–C6 levels (white arrow). **(B, C)** Chest CT scan during the acute phase of SCLS demonstrating bilateral pulmonary interstitial edema and pleural effusion. **(C)** The cervical spinal leision disappeared after six months therapy. **(D, E)** Post-treatment chest CT scan revealing considerable improvement, with only minimal scattered exudative changes and complete resolution of the pleural effusion.

A 20-year-old woman was admitted to our institution in November 2024 with a 1-month history of refractory hiccups and vomiting, along with a 3-week progressive loss of visual acuity in the right eye. Her medical history was unremarkable. On admission, serum testing for AQP-4 antibodies using a cell-based assay revealed a titer of 1:32. Magnetic resonance imaging (MRI) demonstrated abnormal hyperintensities in the spinal cord at levels C2–C6, T4–T5, and T7–T9 (**Figure 1A**), with an absence of significant abnormalities in the brain (**Supplementary Figure S1**). Ophthalmological examination revealed a right pupil measuring 5×5 mm with absent light reflex and no light perception, however the left eye was normal. The orbital MRI revealed abnormal hyperintense signals involving the right pre-chiasmatic optic nerve on T2-weighted fat-suppressed sequences ( **Supplementary Figure S2**).” A diagnosis of NMOSD was established, and the patient’s Expanded Disability Status Scale (EDSS) score was 4. Treatment was promptly initiated with intravenous methylprednisolone 1,000 mg/day for 5 days and five sessions of plasma exchange (PE). Her symptoms substantially improved.

**Figure 2:**
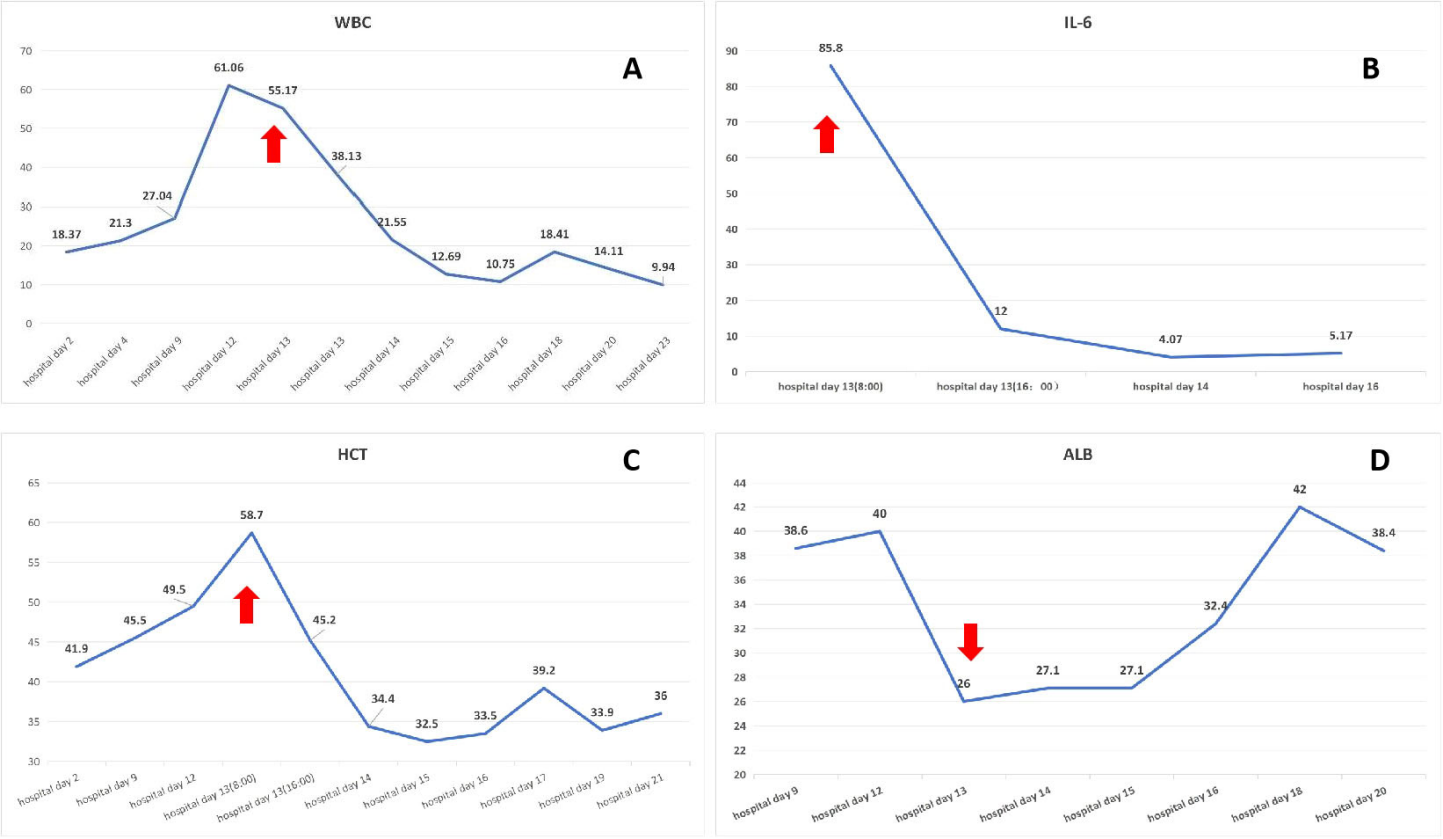
Laboratory values at baseline and during attacks. The patient experienced an acute exacerbation on hospital day 13, with elevated white blood cell count (WBC; **(A)**, 55.17×10^9^/L; reference range: 3.5-9.5×10^9^/L), IL-6 level (**(B)**, 85.8 pg/mL; reference range: <7pg/mL), and hematocrit (**(C)**, 58.7%; reference range: 35%–45%), as well as a decreased serum albumin level (**(D)**, 26 g/L; reference range: 40-50g/L) (red arrow). After treatment, these parameters returned to normal.

On the day of discharge, the patient suddenly developed chest tightness, dyspnea, cold extremities, and loss of consciousness. Vital signs showed severe hypotension (with no measurable blood pressure) and an oxygen saturation of 84%. Laboratory tests revealed leukocytosis, elevated interleukin (IL)-6, and hypoalbuminemia. Hematocrit was 58.7% (reference range: 35%–45%) (**Figure 2**). Liver and kidney function test results were within normal limits. Echocardiography excluded myocardial injury and pericardial effusion. Chest somputed tomography (CT) showed interstitial pulmonary edema and bilateral exudative infiltrates, with a marked increase in pleural effusion compared with the prior study (**Supplementary Figure S3**; **Figures 1B, C**); no clinically significant abnormalities were evident on CT pulmonary angiography. The patient was transferred to the intensive care unit for further management. Pulmonary embolism, cardiogenic shock, and septic shock were excluded as potential diagnoses. Considering the hypotension, hypoalbuminemia, and hemoconcentration (elevated hematocrit) findings, a diagnosis of SCLS was considered.

To investigate the underlying etiology of SCLS, immunoglobulin (Ig) testing was performed, revealing reduced levels of IgG (3.06 g/L; reference range: 8.6–17.4 g/L), IgM (0.32 g/L; reference range: 0.5–2.8 g/L), and IgA (0.79 g/L; reference range: 1.0–4.2 g/L). To exclude monoclonal gammopathy, we performed serum protein electrophoresis with immunofixation (and serum free light-chain assay). No monoclonal band was detected, and the κ/λ ratio was within the reference range, and repeat testing during follow-up showed consistent negative findings. Upon intensive care unit admission, thoracic puncture drainage, blood volume resuscitation, and albumin replacement were promptly initiated. Within 48 hours, the patient’s condition stabilized, as evidenced by resolution of chest tightness and dyspnea. Laboratory results showed a white blood cell count of 12.69×10^9^/L, IL-6 level of 4.07 pg/mL, albumin level of 27.1 g/L, and hematocrit of 33.5%. The patient was subsequently transferred to the general ward.

To prevent recurrence of SCLS, intravenous immunoglobulin (IVIG, 0.4 g/kg/day for 5 days) was administered for hypoimmunoglobulinemia management. Given the elevated IL-6 level, sequential therapy with satralizumab, an IL-6 receptor antagonist, was initiated(subcutaneous administration: 120 mg loading dose at Weeks 0/2/4, 120 mg maintenance dose every 4 weeks; initiated on the 10th day after SCLS acute phase control). Before discharge, the patient visual acuity in the right eye had substantially improved, reaching 20/50 Snellen; fundus examination revealed no definite pathological changes, and the EDSS score decreased to 3.5. Follow-up chest CT demonstrated partial resolution of bilateral exudation and no evidence of pleural effusion (**Figures 2D, E**). IL-6 and albumin levels had normalized (**Figure 2**). At the 13-month follow-up, the patient remained stable, without recurrence of NMOSD or SCLS; her EDSS score was 1.

## Results: literature review and our case

A total of seven articles were reviewed, including one cohort study. Databases included PubMed/MEDLINE, Embase, Web of Science, and regional databases (e.g., CNKI, Wanfang). The search strategy combined terms for systemic capillary leak syndrome (e.g., “SCLS”, “Clarkson disease”) and autoimmune diseases (e.g., “lupus”, “rheumatoid arthritis”, “vasculitis”) using Boolean operators.

In total, 12 patients with comorbid autoimmune diseases and SCLS were identified, including our case (**Table 1**). The male-to-female ratio was 1:3. The mean age at disease onset was 43.7 years (range: 17–86 years). Coexisting autoimmune disorders were as follows: Sjögren’s syndrome (n=5, 41.7%), NMOSD (n=2, 16.7%), chronic inflammatory demyelinating polyradiculoneuropathy (n=1), antiphospholipid syndrome (n=1), polymyositis (n=1), and systemic sclerosis (n=1). Notably, two patients (16.7%) had more than one concurrent autoimmune condition. Infection was the most common trigger (n=4, 33.3%), followed by relapse of the underlying autoimmune disease (n=3, 25%). Other identified triggers included surgical procedures (n=1), physical exertion (n=1), and allergic reactions (n=1). Similar to our case, one NMOSD patient developed SCLS after rituximab administration.

**Table 1:** Clinical characteristics and summary of SCLS cases complicated by autoimmune diseases.

No	Sex	Age (years)	Co-occurring autoimmune diseases	Triggering factors	Disease course(days)	symptoms	Laboratory findings			Antibody	Radiology findings			Immunotherapy	Outcomes
							Hemoglobulin (g/L)	Hematocrit (%)	Albumin (g/L)		CT	X-ray	Ultra-sound		
Chen	Female	20	NMOSD	Flare of autoimmune disease	12	Chest tightness/Dyspnea/Cold extremities/Syncope	165	58.7	26	Anti-aqp4	Bilateral pulmonary interstitial edema and bilateral pleural effusion	NA	normal	Steroid/IVIG/Satralizumab	Alive
Cao (4)	Female	50	Sjögren’s syndrome	Infection	14	Dyspnea	128	42	20	Anti-SSA/Anti-Ro52/ANA	Multiple lung inflammations and bilateral pleural thickening	NA	NA	Steroid/IVIG/CTX	Alive
He (5)	Female	55	Rheumatoid arthritis	Infection	21	Dyspnea/Chest tightness/Edema/Hypotension	92.8	59.38	22.5	ANA/RF	Exudative lesions in both lungs	NA	NA	Steroid/IVIG/MTX/tocilizumab	Alive
Prete (6)	Female	30	Antiphospholipids syndrome	Caesarean section	0.5	Abdominal pains/Dyspnea/Edema	95	29	21	ANA/aPL	Congestion of interstitial pulmonary parenchyma/pericardial and pleural effusion/edema of intestinal wall and of perivisceral adipose tissue, and periportal lymphedema	NA	NA	Steroid/IVIG	Alive
Rao (7)	Male	35	CIDP	None	10	Dyspnea/Hypotension/Respiratory distress	213	62.6	32.8	NA	Ground-glass opacities in both lungs	NA	NA	Steroid/IVIG	Alive
Katsube (8)	Female	86	Rheumatoid arthritis and /vasculitis	Skin ulcers/Infection	2	General edema	107	NA	37	RF	Pelvic computed tomography showed free air just below the abdominal wall and between the small intestine	NA	NA	CTX	Died
Fuentes (9)	Female	61	NMOSD	Used rituximab	30	Edema/Hypotension/Oliguria	186	56.2	10	Anti-aqp4	NA[The autopsy showed only findings of diffuse oedema with ascites and pericardial effusion]	NA	NA	None	Died
Guffroy (10)	Female	25	Sjögren’s syndrome	Infection	15	Edema/Severe weight gain	NA	47.8	15	Anti-SSA/Anti-SSB/ANA	NA	pericarditis	Pericardial effusion/abundant ascites	Steroid/IVIG/HCQ	Alive
Guffroy (10)	Male	45	Sjögren’s syndrome/systemic sclerosis	Flare of autoimmune disease	2	Weight gain/Edema	NA	59.7	25.5	NA	NA	NA	NA	IVIG	Died
Guffroy (10)	Male	17	Polymyositis	Flare of autoimmune disease	15	Edema/Hypotension	NA	57	27.7	None	NA	NA	NA	Steroid/IVIG/MTX/AZA	Alive
Guffroy (10)	Female	45	Sjögren’s syndrome	Physical effort	2	Weakness/Oliguria	NA	56.5	27	Anti-SSA	NA	NA	Pericarditis	IVIG	Alive
Guffroy (10)	Female	55	Sjögren syndrome	Allergy	1	Hypotension	205	64	35	Anti-SSA	NA	NA	NA	Steroid	Alive

CT, computed tomography; NMOSD, neuromyelitis optica spectrum disorders; aqp4, aquaporin-4; NA, not available; IVIG, intravenous immunoglobulin; ANA, antinuclear antibody; CTX, cytoxan; RF, rheumatoid factors; MTX, methotrexate; aPL, antiphospholipid antibody; CIDP, chronic inflammatory demyelinating polyneuropathy; HCQ, hydroxychloroquine; AZA, azathioprine.

Patients typically presented with acute symptom onset; the mean interval between the appearance of prodromal symptoms and onset of SCLS was average 10.4 days (range: 0.5–30 days). Cardiovascular involvement was the most frequent clinical presentation, followed by respiratory symptoms. Laboratory findings revealed a mean hemoglobin level of 149.0g/L (range: 92.8-213.0g/L), hematocrit of 53.9% (range: 29-64%), and serum albumin level of 24.9g/L (range: 10-37g/L). Autoantibodies were detected in nine patients, including anti-SSA/SSB antibodies (n=4), antinuclear antibodies (n=3), anti–AQP-4 antibodies (n=2), antiphospholipid antibodies (n=1), anti-Ro52 antibodies (n=1), and rheumatoid factor (n=1). Pulmonary and abdominal CT scans demonstrated interstitial pulmonary edema, pleural effusion, pelvic effusion, intestinal wall edema, and lymphadenopathy. Ultrasonographic evaluations identified pericardial effusion in two patients.

Three distinct treatment strategies were identified in this cohort. The primary therapeutic regimen, consisting of glucocorticoids combined with IVIG, was administered to seven patients (58.3%). Among these, four patients required additional immunomodulatory therapy: three received conventional immunosuppressants (cyclophosphamide, methotrexate, and hydroxychloroquine), whereas two were treated with biologic agents (tocilizumab and satralizumab). Monotherapy regimens demonstrated limited efficacy; four patients (33.3%) received single-agent treatments, including glucocorticoids alone (n=1), IVIG monotherapy (n=2), and cyclophosphamide monotherapy (n=1). Notably, one patient died because of rapid disease progression before immunotherapy initiation. Clinical improvement was observed in nine patients (75.0%) after aggressive multimodal therapy; three patients (25.0%) died. Among the deceased, two (66.7%) were aged ≥60 years, and one (33.3%) had polyautoimmunity (concurrent Sjögren’s syndrome and systemic sclerosis).

## Discussion

SCLS is a rare and potentially fatal condition characterized by episodic hypovolemic shock, generalized edema, hemoconcentration, and hypoalbuminemia. It was first described in 1960 by Clarkson et al. (6, 11). To date, more than 300 cases of SCLS have been reported worldwide (12). The etiology of SCLS remains incompletely understood. In approximately 80% of cases, a monoclonal component can be identified. However, sepsis, cardiac surgery (13), anaphylaxis, and severe burns have also been reported to provoke SCLS-like manifestations (14–17). Certain medications, including antineoplastic and immunomodulatory agents, have been implicated as contributing factors (18, 19).

Autoimmune diseases are common comorbidities of SCLS. Our review indicated that overlap between autoimmune diseases and SCLS can occur across all age groups, although it predominantly affects young to middle-aged adults and exhibits a strong female predominance. Systemic autoimmune disorders, particularly Sjögren’s syndrome, and neurological conditions such as NMOSD are frequently associated with this syndrome. The clinical course is typically acute and often triggered by infections or autoimmune disease flares. Circulatory and respiratory compromise are the primary clinical manifestations, accompanied by hallmark laboratory findings of hemoconcentration and hypoalbuminemia (20). Diagnosis is based on characteristic imaging findings and the exclusion of other causes of increased vascular permeability. Notably, our case patient was diagnosed with NMOSD 1 month after symptom onset, with immunotherapy initiated thereafter. At onset, multifocal neurological involvement was documented, and the EDSS score exceeded 3.0. Whether delayed therapeutic intervention and the high severity of disease are directly associated with the development of SCLS in patients remains to be elucidated.

The mechanisms linking autoimmune diseases and SCLS remain incompletely understood. However, studies have shown that inflammation can induce endothelial cell activation, characterized by the release of proteins such as P-selectin, von Willebrand factor, ILs, and angiopoietin-2 from intracellular Weibel–Palade bodies. This activation promotes increased capillary permeability, facilitating immune cell trafficking and macromolecule extravasation at sites of localized inflammation (21). When this pro-inflammatory response becomes systemic, widespread endothelial barrier dysfunction can occur, resulting in SCLS (22). In our case, the patient exhibited substantially elevated serum IL-6 levels during the acute phase of SCLS. Studies have also demonstrated that IL-6 levels are considerably increased in the peripheral blood of NMOSD patients. IL-6 has been shown to reduce the expression of endothelial tight junction proteins in a dose- and time-dependent manner, leading to increased permeability among human brain microvascular endothelial cells (23). Whether IL-6 acts as a shared pathogenic factor for both NMOSD and SCLS, however, remains to be determined.

In the reviewed literature, 58.3% of patients with autoimmune disease-associated SCLS were treated with glucocorticoids combined with IVIG, which remains a cornerstone of SCLS management for its proven efficacy in reducing the frequency and severity of acute attacks (24, 25). After resolution of acute SCLS manifestations, long-term immunomodulatory therapy should be individualized per the underlying autoimmune disorder. Notably, our literature review identified a case of rheumatoid vasculitis in which tocilizumab, an anti-IL-6 receptor agent, was administered as disease-modifying therapy post SCLS remission, leading to sustained control of both conditions (5)—a finding that highlights the value of targeted immunoregulation for secondary prevention of SCLS in autoimmune diseases. In the present case, following stabilization of acute SCLS with multimodal supportive and immunotherapeutic interventions (albumin replacement, thoracic drainage and IVIG), maintenance therapy with satralizumab, a humanized anti-IL-6 receptor monoclonal antibody, was initiated. The SAkuraStar trial (NCT02073279) confirmed that satralizumab significantly reduces relapse rates in AQP-4-IgG seropositive NMOSD patients (26), and we hypothesized that this agent may also contribute to SCLS recurrence prevention by inhibiting IL-6 to preserve vascular endothelial integrity (27, 28). Over more than one year of satralizumab treatment in conjunction with standard NMOSD management, the patient achieved a sustained reduction in EDSS score to 1, with no clinical or radiological evidence of NMOSD or SCLS recurrence. While this favorable outcome was observed during satralizumab therapy, the multifactorial nature of the initial intervention (including IVIG and glucocorticoids) and the lack of a controlled design preclude definitive attribution of the sustained remission solely to IL-6 blockade. This study has several limitations that merit acknowledgment: (1) potential selection bias in the literature review, such as publication bias toward severe cases; (2) heterogeneous SCLS diagnostic criteria across included studies; (3) the inability to establish a causal relationship between autoimmune diseases and SCLS; and (4) limited follow-up data for the study cohort. This report highlights a rare case of NMOSD co-occurring with SCLS, providing important clinical insights into the association between SCLS and autoimmune diseases. Further in-depth studies are needed to elucidate the complex relationship between these two conditions and evaluate therapeutic efficacy. Moreover, IL-6 may represent a promising therapeutic target, and satralizumab could warrant further investigation as a potential treatment option within a comprehensive management strategy in such cases.

## Data Availability

The raw data supporting the conclusions of this article will be made available by the authors, without undue reservation.

## Supplementary material

Supplementary Figure 1 Cerebral MRI of the patient. No abnormal signal intensity was identified in the dorsal medulla oblongata on the patient’s axial T2-weighted imaging.

Supplementary Figure 2 Axial MRI sequences of the optic chiasm in our patient. **(A)** T2-weighted, **(B)** T2-weighted fat-suppressed, and **(C)** T1-weighted post-contrast images. Red arrows indicate focal hyperintensity and edema at the right pre-chiasmatic optic nerve in **(A)** and **(B)**, with corresponding contrast enhancement in **(C)**. These findings are consistent with inflammatory demyelination and correlate with the patient’s right eye blindness.

Supplementary Figure 3 Chest CT scan obtained 1 day prior to the onset of SCLS in the patient, revealing bilateral interstitial pulmonary edema, bilateral exudative infiltrates **(A)**, and a small pleural effusion **(B)**.
