# Supplementary figures and images for: Case Report: Rare co-occurrence of NMOSD and capillary leak syndrome treated with satralizumab
Source: Front Immunol. 2026 Mar 4;17:1684380. doi: 10.3389/fimmu.2026.1684380 (PMC12996117; doi:10.3389/fimmu.2026.1684380)

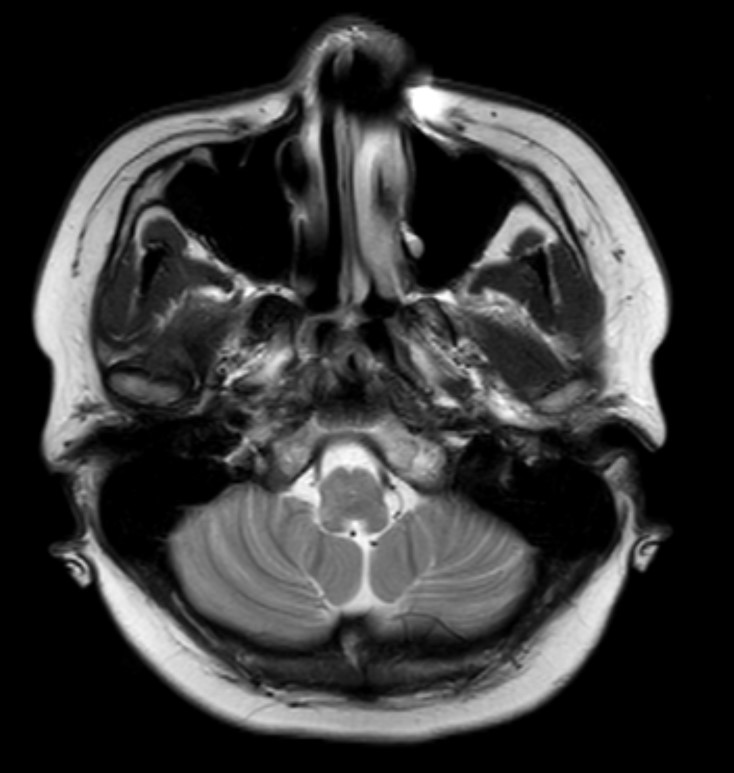

Supplement: Supplementary Figure 1 — Cerebral MRI of the patient. No abnormal signal intensity was identified in the dorsal medulla oblongata on the patient’s axial T2-weighted imaging. [file Image1.jpeg]

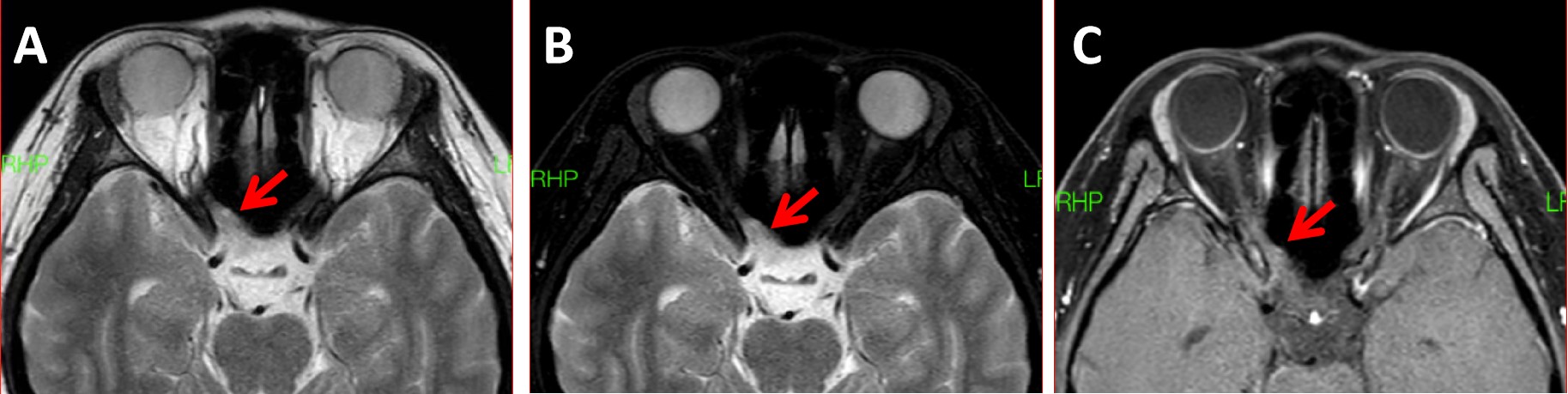

Supplement: Supplementary Figure 2 — Axial MRI sequences of the optic chiasm in our patient. (A) T2-weighted, (B) T2-weighted fat-suppressed, and (C) T1-weighted post-contrast images. Red arrows indicate focal hyperintensity and edema at the right pre-chiasmatic optic nerve in (A) and (B), with corresponding contrast enhancement in (C). These findings are consistent with inflammatory demyelination and correlate with the patient’s right eye blindness. [file Image2.jpeg]

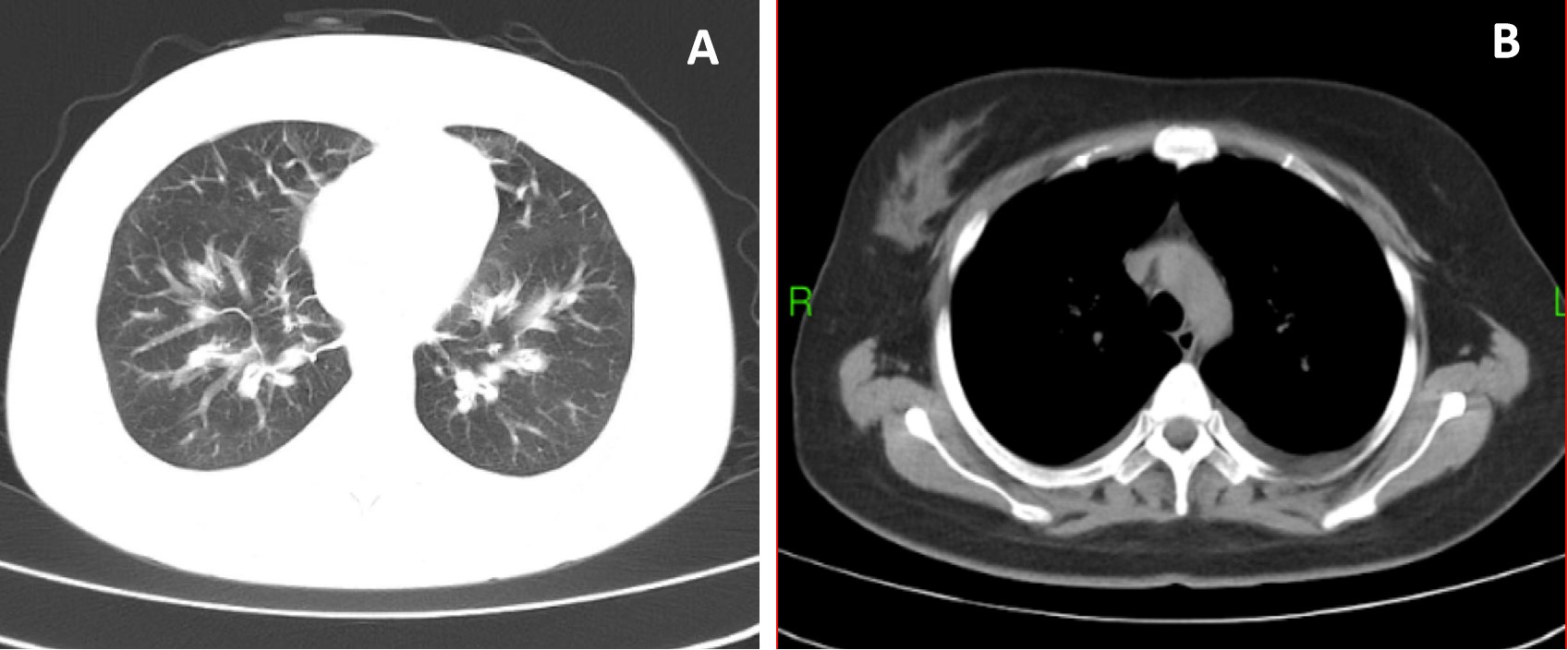

Supplement: Supplementary Figure 3 — Chest CT scan obtained 1 day prior to the onset of SCLS in the patient, revealing bilateral interstitial pulmonary edema, bilateral exudative infiltrates (A), and a small pleural effusion (B). [file Image3.jpeg]
